# Effects of Wenxin Keli on Cardiac Hypertrophy and Arrhythmia via Regulation of the Calcium/Calmodulin Dependent Kinase II Signaling Pathway

**DOI:** 10.1155/2017/1569235

**Published:** 2017-05-09

**Authors:** Xinyu Yang, Yu Chen, Yanda Li, Xiaomeng Ren, Yanwei Xing, Hongcai Shang

**Affiliations:** ^1^The Key Laboratory of Chinese Internal Medicine of the Ministry of Education, Dongzhimen Hospital Affiliated to Beijing University of Chinese Medicine, Beijing 100700, China; ^2^Guang'anmen Hospital, Chinese Academy of Chinese Medical Sciences, Beijing 100053, China; ^3^Fujian Health College, Fuzhou 350101, China

## Abstract

We investigated the effects of Wenxin Keli (WXKL) on the Calcium/Calmodulin dependent kinase II (CaMK II) signal transduction pathway with transverse aortic constriction (TAC) rats. Echocardiographic measurements were obtained 3 and 9 weeks after the surgery. Meanwhile, the action potentials (APDs) were recorded using the whole-cell patch clamp technique, and western blotting was used to assess components of the CaMK II signal transduction pathway. At both 3 and 9 weeks after treatment, the fractional shortening (FS%) increased in the WXKL group compared with the TAC group. The APD_90_ of the TAC group was longer than that of the Sham group and was markedly shortened by WXKL treatment. Western blotting results showed that the protein expressions of CaMK II, phospholamban (PLB), and ryanodine receptor 2 (RYR2) were not statistically significant among the different groups at both treatment time points. However, WXKL treatment decreased the protein level and phosphorylation of CaMK II (Thr-286) and increased the protein level and phosphorylation of PLB (Thr-17) and the phosphorylation of RYR2 (Ser-2814). WXKL also decreased the accumulation of type III collagen fibers. In conclusion, WXKL may improve cardiac function and inhibit the arrhythmia by regulating the CaMK II signal transduction pathway.

## 1. Introduction

Cardiovascular diseases are the most common threat to human health worldwide and are the leading cause of morbidity among humans. In many cardiovascular diseases, cardiac hypertrophy is a common pathological process, and the cardiac arrhythmia induced by it is the most common cause of sudden cardiovascular death. Cardiac hypertrophy is a maladaptive change in response to pressure overload and is also an important risk factor for developing heart failure [1]. Pathological hypertrophy is characterized by significant changes in the size, shape, wall thickness, and contractile function of the cardiac chamber [2, 3]. At the level of single cardiomyocytes, hypertrophy is simply defined as an increase in the cardiomyocyte size. A previous study in a cohort of 690 athletes has found that 36% of the athletes died as a result of cardiac hypertrophy [4]. These results highlight the importance of finding suitable agents for treating cardiac hypertrophy.

CaMK II belongs to the subfamily of multifunctional Ser/Thr kinases, which phosphorylate a variety of substrates and regulate numerous cellular functions [5–8] that are intimately involved in heart diseases [9–11]. Activation of CaMK II is an important step in the signaling of cardiac hypertrophy. Several studies have demonstrated that CaMK II plays important functions in the development of cardiac hypertrophy by causing impaired gene expression [12]. Increasing understanding of CaMK II and its effects on the heart lends support to its potential as a therapeutic target [13]. The compound KN93 (2-[N-(2-hydroxyethyl)]-N-(4-methoxybenzenesulfonyl)amino-N-(4-chlorocinnamyl)-N-methylbenzylamine) has been widely used as a pharmacological tool to inhibit CaMK II in several studies [14–16].

Wenxin Keli (WXKL) is a Chinese herbal extract developed at the Guang'anmen Hospital of the Chinese Academy of Chinese Medical Sciences, and it is the first Chinese medicine to be approved by the Chinese state for use in arrhythmia. The main ingredients of WXKL consist of* Nardostachys chinensis Batal*,* Codonopsis*,* notoginseng*,* amber*, and* Rhizoma Polygonati*. A large number of clinical trials have confirmed that WXKL can increase coronary blood flow, decrease myocardial oxygen consumption, enhance myocardial compliance, improve myocardial hypoxia tolerance, relieve anterior and posterior cardiac loading, decrease myocardial tissue damage in patients with high blood pressure, and decrease the occurrence of arrhythmia [17]. This drug has been proven to be beneficial in the treatment of various diseases, such as cardiac arrhythmias, cardiac inflammation, cardiac hypertrophy, and chronic heart failure [18]. In the present study, we sought to determine whether WXKL and KN93 decrease cardiac hypertrophy and arrhythmia by regulating the CaMK II signal transduction pathway.

## 2. Materials and Methods

### 2.1. WXKL

WXKL, consisting of* Rhizoma Nardostachys*,* notoginseng*,* Codonopsis*,* amber*, and* Rhizoma Polygonati*, was provided by the BuChang Group, Shandong, China. According to the national pharmacopoeia (National Pharmacopoeia Committee, 2005), the total amount of Ginseng saponin Rg1 (C_42_H_72_O_14_),* notoginseng saponin R1* (C_47_H_80_O_18_), and* Ginseng saponin Rb1* (C_54_H_92_O_23_) should not be less than 17 mg per bag (9 g). The powdered WXKL compound was dissolved in distilled water before use.

### 2.2. Animal Groups and Administration of Drugs

One hundred male Sprague-Dawley rats (body weight: 140–160 g), purchased from the Vital River Experimental Animal Center (Beijing, China), were randomly divided into two groups: the TAC group (*n* = 75) and the Sham group (*n* = 25) by using a random number table. The TAC rats underwent transverse aortic constriction surgery, and those in the Sham group underwent an identical procedure but without the application of ligation. The 75 TAC rats were randomly assigned to three treatment groups by using a random number table after ultrasonic cardiogram evaluation: the TAC group (*n* = 25), in which the rats were treated with the vehicle alone (distilled water, 1 mL/kg/day) by oral administration; the WXKL group (*n* = 25), in which the rats were treated with the WXKL compound (4 g/kg/day) by oral administration; and the KN93 group (*n* = 25), in which the rats were treated with KN93 (14 *μ*mol/kg/day) by intraperitoneal injection. After 3 weeks of treatment, 10 rats were randomly selected from each group for the experiment. After 9 weeks of treatment, there were 15 rats in the Sham group, 10 rats in the TAC group (5 rats died), 13 rats in the KN93 group (2 rats died), and 12 rats in the WXKL group (3 rats died). All animals used in this study received humane care in compliance with the National Institutes of Health Guide for the Care and Use of Laboratory Animals.

### 2.3. Establishment of the TAC Model and Sham-Operated Rats

The TAC surgery was performed in male Sprague-Dawley rats as described previously [19, 20]. Briefly, the rats were anaesthetized by intraperitoneal injection of a 3% solution of chloral hydrate (300 mg/kg). The preparation process included endotracheal intubation, positive pressure ventilation, and preoperative recording by twelve-lead ECG. Then, the thorax was opened, and a 4-0 silk suture was passed under the aorta between the origins of the right innominate and the left common carotid arteries. A 6G needle was placed on the ascending aorta, and the suture was snugly tied around the needle and the aorta. The probe was then quickly removed. The skin was closed, and the rats were maintained on a heating pad until they recovered from the anesthesia. The Sham-operated animals underwent an identical procedure but without the application of ligation. After surgery, both groups were given tap water and normal chow in different cages. To characterize the model, echocardiographic measurements were obtained 3 and 9 weeks after the surgery using a Vivid 7 Dimension cardiovascular ultrasound system (GE Healthcare, Fairfield, Connecticut, United States) as described previously [21]. The indexes for the left ventricular posterior wall thickness (LVPWS), left ventricular internal dimension systole (LVIDs), and FS% were shown in Figure 2.

### 2.4. Histological Examination

Rat heart samples were cut into transverse sections and stained with hematoxylin and eosin (H&E), Masson's trichrome, and Sirius Red dye as described previously [22]. The stained sections were examined under a light microscope (OLYMPUS BX51, Japan) and photographed at 400x magnification for morphological analysis. Meanwhile, the rat myocardial cells were processed for transmission electron microscopy (H-600, Japan) according to routine procedures, as previously described [23].

### 2.5. Isolation of Cardiac Ventricular Myocytes

Single cardiac ventricular myocytes were isolated from the hearts of the rats as previously described [24] with slight modifications. Briefly, 5 minutes after the rats were heparinized (100 U/mL 1 mL/100 g i.p.), the animals were anesthetized with 3% chloral hydrate (0.5 mL/100 g i.p.). The hearts were rapidly excised and mounted on the Langendorff apparatus and perfused via the aorta with oxygenated Ca^2+^-free Tyrode solution for 5 minutes and then with Ca^2+^-free Tyrode solution containing collagenase II (0.6 mg/mL, Worthington, USA), trypsin (0.24 mg/mL, Amresco, USA), and proteinase E (0.08 mg/mL, Amresco, USA) for 15–20 minutes at 37°C. Subsequently, the ventricular tissue was excised, cut into small pieces in a dish containing KB solution, and blown gently to obtain single ventricular myocytes. The cells were maintained at 4°C in the KB solution until use. All the solutions were continuously gassed with 95% O_2_ and 5% CO_2_ at 37°C. The single ventricular myocytes selected for electrophysiological measurements were rod-shaped, quiescent, and Ca-tolerant and had clear cross-striations with a smooth and glossy surface.

### 2.6. Electrophysiological Recording

The whole-cell patch clamp technique was used to record the APs using an Axopatch 700B amplifier (Axon Instruments, USA) and the data were analyzed with pCLAMP 9.2 software (Axon Instruments, USA). Borosilicate glass patch pipettes (resistance = 3–5 MΩ) were pulled using a vertical pipette puller (Narishige pp-830, Japan). The cells were maintained in an external solution for 5 to 10 minutes after perfusion, and the data were recorded after piercing the cell for 5 minutes to stabilize the current. Data recordings were performed at room temperature (22°C) within 25 minutes to avoid current rundown. APs were initiated in the current clamp mode at a rate of 1.0 Hz using 30 trains of suprathreshold current pulses. Membrane capacitance was calculated using the manual whole-cell capacitance controls on the Axopatch amplifier.

### 2.7. Western Blot Analysis

The animals were euthanized after 3 weeks and 9 weeks of drug administration, and their hearts were immediately harvested and stored in liquid nitrogen until western blot analyses were performed. The antibodies to the following proteins were used: CaMK II delta (1 : 1000, Abcam Corporation.), phospho-CaMK II (1 : 1000, Cell Signaling Technology Inc.), PLB (G-18) (1 : 200, Santa Cruz Biotechnology Inc.), p-phospholamban-R (1 : 200, Santa Cruz Biotechnology Inc.), and ryanodine receptor 2 (1 : 1000, Millipore Corporation.). The proteins were separated by 10% SDS-PAGE and transferred onto nitrocellulose membranes, which were then incubated with antibodies at 4°C. The membranes were further incubated for 2 hours at room temperature. ECL visualization was performed, and a GeneGnome Gel Imaging System (Syngene Co.) was used to capture the resulting images. ImageJ (Image-Pro plus analysis software) was used to analyze the gel images.

### 2.8. Statistical Analyses

All experimental data were expressed as the means ± SD. The data were statistically evaluated using one-way analysis of variance (ANOVA), and a post hoc analysis was performed using Fisher's least significant difference (LSD) test. The SPSS program (version 20.0) was used for the analyses. A probability of *P* < 0.05 was considered statistically significant. The pCLAMP 9.2 software (Axon Instruments, USA) and Origin 6.1 software (MicroCal Software, USA) were used for the data acquisition and analysis.

## 3. Results

### 3.1. Effects of Treatment with WXKL and KN93 for 3 and 9 Weeks on Cardiac Morphology

**Figure 1:**
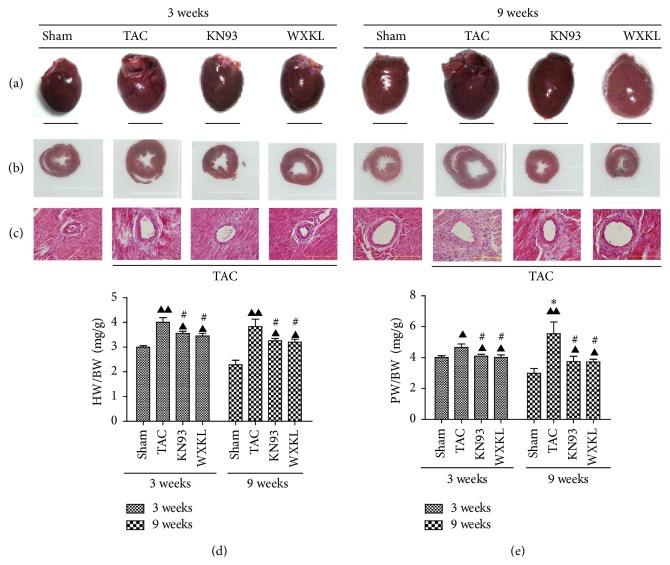
(a) Representative images of hearts from the Sham (*n* = 5), TAC (*n* = 5), KN93 (*n* = 5), and WXKL (*n* = 5) groups at 3 and 9 weeks after treatment. Scale: 1 cm. (b) Representative images of the largest cross-section of the heart from each group at 3 and 9 weeks (*n* = 5). (c) Representative images of left ventricular apical biopsy of each group at 3 and 9 weeks (H&E staining at 400x magnification; *n* = 5). (d) Ratios of heart weight to body weight (HW/BW) are shown (*n* = 5). (e) Ratios of pulmonary weight to body weight (PW/BW) are shown at 3 and 9 weeks. (*n* = 5). ^▲^*P* < 0.05 and ^▲▲^*P* < 0.01 versus the Sham group. ^#^*P* < 0.05 versus the TAC group. ^*∗*^*P* < 0.05 versus the TAC group at 3 weeks.

The extent of myocardial fibrosis was improved in the KN93 and WXKL groups compared with the TAC group after 3 and 9 weeks of treatment (Figure 1(c), HE staining 400x magnification). At 3 and 9 weeks, the ratios of heart weight to body weight (HW/BW) of the TAC group were significantly higher than those of the Sham group, but those of the KN93 and WXKL groups were less than the values from the TAC group (Figure 1(d)). Meanwhile, the ratios of pulmonary weight to body weight (PW/BW) of TAC group were only slightly higher than those of the Sham group, but the ratios from the KN93 and WXKL groups were significantly lower than those of the TAC group (Figure 1(e)). Overall, the values for PW/BW at 9 weeks were significantly higher than those at 3 weeks.

### 3.2. Assessment of Cardiac Function by Echocardiography

**Figure 2:**
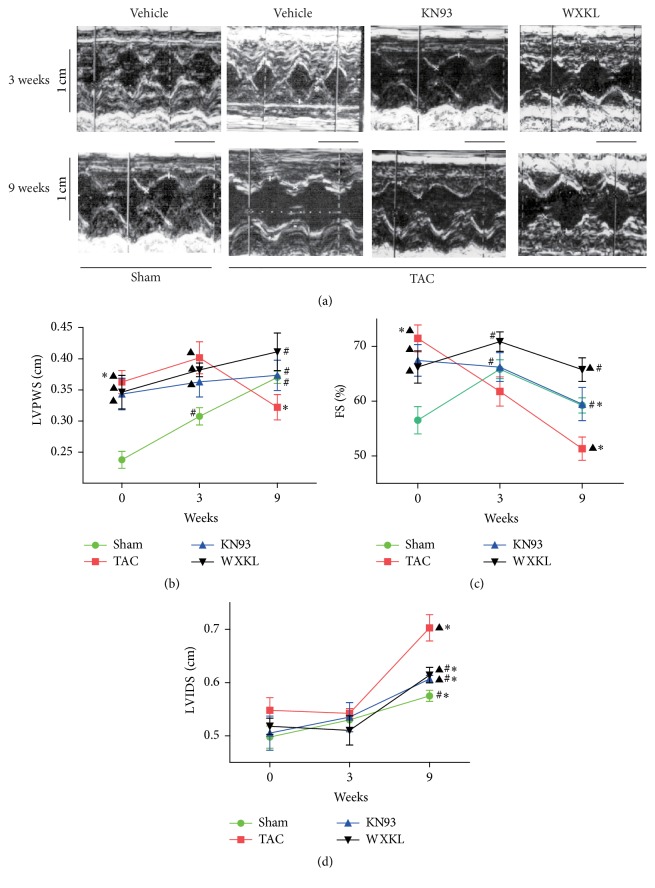
(a) Typical echocardiography images from the Sham (*n* = 10), TAC (*n* = 10), KN93 (*n* = 10), and WXKL (*n* = 10) groups at 3 and 9 weeks. Scale: 0.5 cm. (b) LVPWS of each group (*n* = 10). (c) FS% of each group (*n* = 10). (d) LVIDs of each group (*n* = 10). ^▲^*P* < 0.05 versus the Sham group. ^#^*P* < 0.05 versus the TAC group. ^*∗*^*P* < 0.05 versus corresponding values at 3 weeks.

We evaluated cardiac function by a combination of echocardiography measurements that included the LVPWS, FS%, and LVIDs (Figure 2). Before treatment, compared with the Sham group, the LVPWS of the TAC, WXKL, and KN93 groups were significantly higher. After treatment for 3 weeks, the LVPWS of the KN93 and WXKL groups were not different from that of the TAC group. Interestingly, after treatment for 9 weeks, the LVPWS became significantly thicker in the KN93 and WXKL groups compared with the TAC group (Figure 2(b)). After treatment for 3 weeks, the FS% decreased in the TAC group compared with the Sham group, but it increased in the WXKL group compared with the TAC group. After 9 weeks, the FS% in the KN93 and WXKL groups was significantly higher than that in the TAC group (Figure 2(c)). However, the LVIDs of the TAC group had no significant difference compared with those of the KN93 and WXKL groups after 3 weeks of treatment, but the LVIDs decreased in the KN93 and WXKL groups compared with the TAC group after treatment after 9 weeks of treatment (Figure 2(d)).

### 3.3. Effects of WXKL and KN93 on Myocardial Cell Morphology in a Rat Model of Cardiac Hypertrophy Induced by TAC

**Figure 3:**
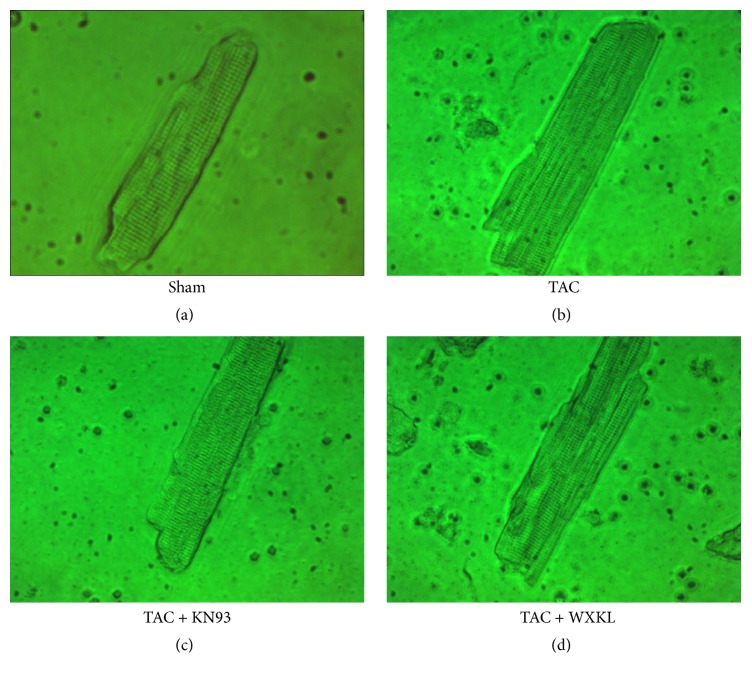
Single ventricular myocytes from the Sham (a), TAC (b), KN93 (c), and WXKL (d) group (400x).

After 9 weeks of treatment, we isolated single ventricular myocytes through enzymatic hydrolysis. After allowing the cells to adhere to slides, the cells with neat edges and clear stripes were observed under a microscope. Compared with those in the Sham group, the single ventricular myocytes in the TAC group were larger (Figure 3). In addition, in the KN93 and WXKL groups, the cell morphology was improved, and the degree of myocardial hypertrophy was decreased compared with that in the TAC group.

### 3.4. Changes in the Microstructure of the Myocardial Tissue in a Rat Model of Cardiac Hypertrophy Induced by TAC

**Figure 4:**
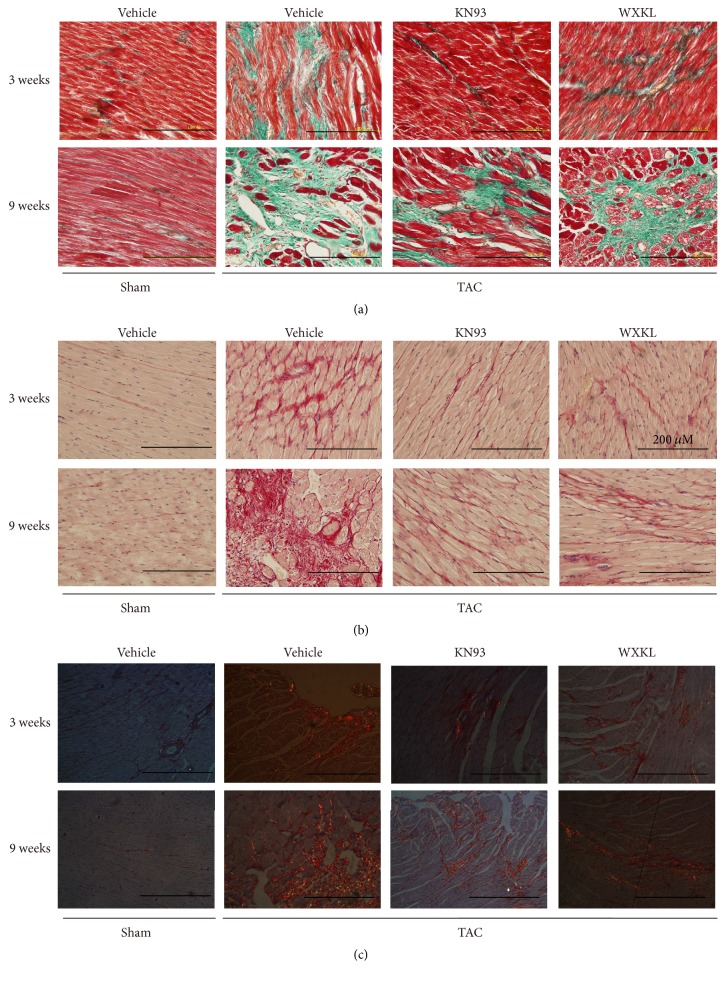
Representative images of (a) Masson's trichrome staining method to evaluate cardiac fibrosis of each group at 3 and 9 weeks (*n* = 5), (b) Sirius Red staining to evaluate cardiac fibrosis of each group at 3 and 9 weeks (*n* = 5), and (c) Sirius Red staining to evaluate cardiac fibrosis of each group at 3 and 9 weeks under polarized light (*n* = 5).

Stained sections were observed under a light microscope, and photos were taken under 400x magnification. Collagen deposition was observed under ordinary light microscope. The results of Masson's trichrome staining showed that the collagen fibers were stained green. The cytoplasm and muscle fibers were stained red. The red blood cells were stained orange, and the nuclei were stained blue-brown (Figure 4(a)). In the Sirius Red staining, the collagen fibers were stained red, and the nuclei were stained blue (Figure 4(b)).

After treatment for 3 weeks, Masson's trichrome staining showed that the TAC group had fibrosis, and collagen deposition was apparent. In addition, after treatment for 9 weeks, collagen deposition in the TAC group was even more pronounced, and the degree of heart failure was significantly increased. In contrast, in the KN93 and WXKL groups, the extent of collagen deposition was greatly decreased (Figure 4(a)). As viewed under a light microscope, the red staining showed that the degree of fibrosis was the most intense in the TAC group. However, fibrosis in the WXKL group was significantly decreased at 9 weeks (Figure 4(b)). Under a polarized light microscope, Sirius Red stained the type I collagen in red or yellow and the type III collagen in green. Sirius Red-stained sections clearly displayed the type I and type III collagen fibers (Figure 4(c)). The results showed that the type I collagen fibers were arranged closely, showing strong refraction, and they appeared bright orange red or bright yellow. In contrast, the staining for type III collagen fibers was very weak and not as apparent.

### 3.5. Ultrastructural Changes in Cardiac Myocytes in a Rat Model of Cardiac Hypertrophy Induced by TAC

**Figure 5:**
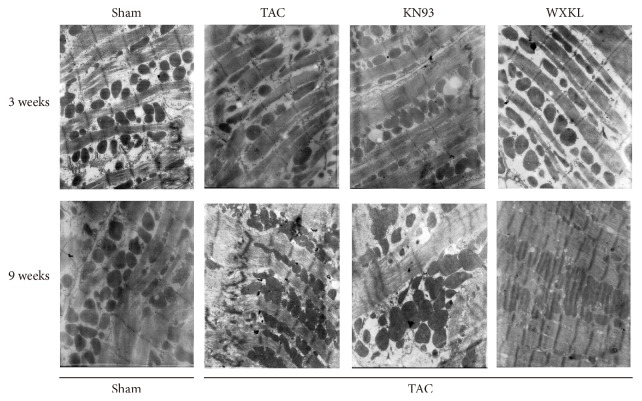
The ultrastructure of myocardial cells in each group at 3 and 9 weeks (*n* = 5) (Magnification 10,000x).

After treatment for 3 weeks, analysis of the ultrastructure of cardiac myocytes showed that the myocardial cells of the Sham group were arranged in a compact manner, and the muscle sections were clear and complete. The mitochondrial structure was normal, and there were abundant glycogen granules. However, in the TAC group, many of the cardiac muscle cells had been dissolved, and some gaps appeared. The mitochondria were swollen, and the ridges were broken. After KN93 and WXKL drug treatment, some myocardial cells had been dissolved, the mitochondria still appeared swollen, and the ridges were still broken. After treatment for 9 weeks, in the Sham group, the myocardial cells were basically complete, and the arrangement of the filaments was more regular. The structure of the mitochondria was also normal. Once again, in the TAC group, the myocardial cells were dissolved to a much greater extent, and their arrangement was more disordered. The morphology of the mitochondria was also substantially altered, and the crest had been fractured. However, after KN93 and WXKL drug treatment, the myocardial cells showed slight recovery, but there was still a significant change in the morphology of mitochondria (Figure 5).

### 3.6. Effects of WXKL and KN93 on the APs after Treatment for 3 and 9 Weeks

**Figure 6:**
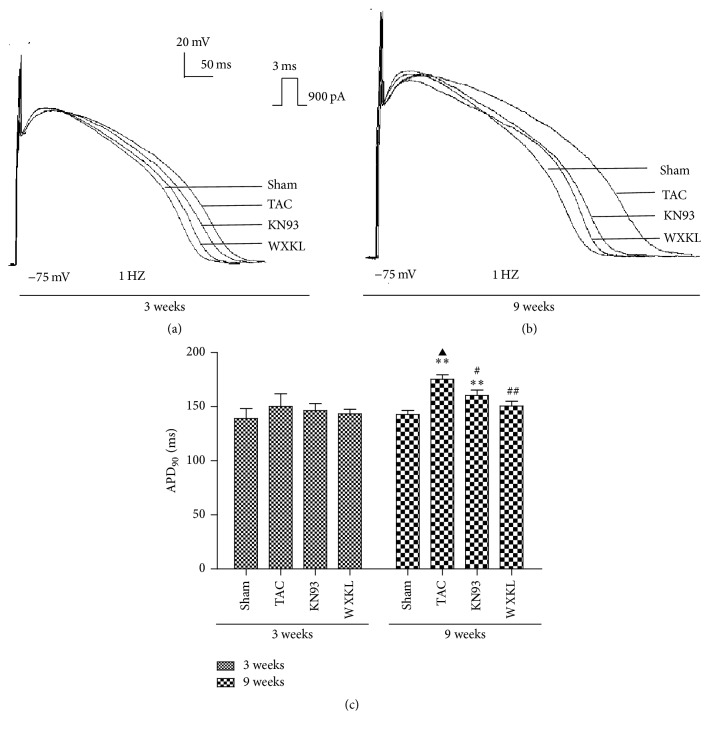
Representative AP traces recorded from each group. (a) APs of each group treated for 3 weeks (*n* = 5). (b) APs of each group treated for 9 weeks (*n* = 5). (c) APD90 of each group after treatment with KN93 and 5 g/L WXKL for 3 weeks and 9 weeks (*n* = 5). ^*∗∗*^*P* < 0.01 versus the Sham group. ^#^*P* < 0.05 and ^##^*P* < 0.01 versus the TAC group. ^▲^*P* < 0.05 versus the TAC group after 3 weeks.

The APs were recorded by application of a 900-pA current pulse with a duration of 3 ms at 1 Hz in the current clamp mode. After treatment for 3 weeks, the APD_90_ was slightly prolonged in the TAC group compared with that in the Sham group (Figure 6(a)). The APD_90_ values of the Sham and TAC groups were 139.0 ± 9.3 ms and 150.3 ± 11.6 ms, respectively (Figure 6(c)). After treatment with KN93 and WXKL, the APD_90_ changed slightly to 146.4 ± 6.5 ms and 143.4 ± 4.1 ms, respectively. The WXKL group thus showed a shortened APD_90_. After treatment for 9 weeks, the APD_90_ was significantly prolonged in the TAC group compared with the Sham group (Figure 6(b)). APD_90_ of the Sham and TAC groups were 142.7 ± 3.9 ms and 175.4 ± 4.2 s, respectively (Figure 6(c)). After treatment with KN93 and WXKL, the APD_90_ changed significantly to 160.4 ± 5.0 ms, and 150.5 ± 4.6 ms. The results thus showed that WXKL and KN93 shortened the APD_90_.

### 3.7. Effects of WXKL and KN93 on the Expression of CaMK II and Related Proteins

**Figure 7:**
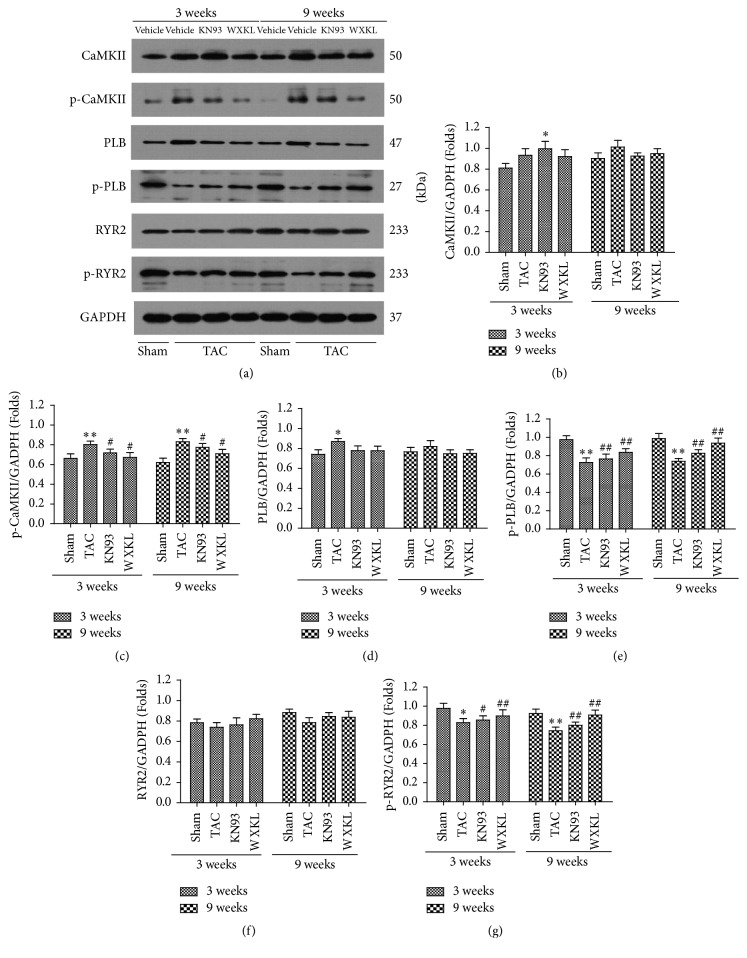
(a) The expression of CaMK II and related proteins in the CaMK II signal transduction pathway in the left ventricular areas after treatment for 3 and 9 weeks were analyzed by western blotting. (b) The expression of CaMK II (*n* = 5). (c) The expression of p-CaMK II (Thr-286) (*n* = 5). (d) The expression of PLB (*n* = 5). (e) The expression of p-PLB (Thr-17) (*n* = 5). (f) The expression of RyR2 (*n* = 5). (g) The expression of p-RyR2 (Ser-2814) (*n* = 5). ^*∗*^*P* < 0.05 and ^*∗∗*^*P* < 0.01 versus the Sham group. ^#^*P* < 0.05 and ^##^*P* < 0.01 versus the TAC group.

Western blotting analysis was performed to examine the expression of CaMK II, Thr-286, PLB, Thr-17, RYR2, and Ser-2814 in the left ventricular apex among the four experimental groups (Figure 7(a)).

After treatment for 3 and for 9 weeks, the difference in the expression of CaMK II was not statistically significant among any of the groups (Figure 7(b)). However, the level of Thr-286 was increased in the TAC group compared with that in the Sham group. The levels of Thr-286 in the WXKL and KN93 groups were significantly decreased compared to in the TAC group (Figure 7(c)). There was a similar trend in the change after treatment both for 3 weeks and for 9 weeks on the levels of Thr-286.

The protein expression of PLB was not statistically significant after treatment for 9 weeks (Figure 7(d)). At both treatment time points, the protein expression of Thr-17 was significantly decreased in the TAC group compared with the Sham group. However, the levels of Thr-17 in the WXKL and KN93 groups were increased after treatment (Figure 7(e)).

At both 3 and 9 weeks after treatment, the difference in the level of RyR2 protein expression was not statistically significant among the different groups (Figure 7(f)). At both treatment time points, however, the levels of Ser-2814 were decreased in the TAC group compared with the Sham group, whereas it was higher than in the TAC group in the WXKL and KN93 groups (Figure 7(g)).

## 4. Discussion

This study revealed that WXKL and KN93 significantly decreased the development of cardiac hypertrophy, thus suggesting that WXKL and KN93 preserve cardiac function after TAC in rats. Our results also showed that the beneficial effects of WXKL and KN93 might be attributed to the shortening of APD_90_ and regulation of the CaMK II signal transduction pathway, thus eventually improving cardiac electrical remodeling and tissue remodeling in hypertrophic cardiac myocytes.

Cardiac hypertrophy is a common pathological change that increases the incidence and mortality in many cardiovascular diseases. These changes are frequently induced by electrical remodeling and genesis of arrhythmia. The results from histology and immunohistochemistry analyses showed that WXKL and KN93 had a significant effect on the hypertrophic myocardium. H&E and Masson's trichrome staining showed that the cardiomyocytes in the TAC group were significantly larger and more loosely arranged. Cardiac fibrosis is a common response of the heart to many forms of injury and is the key pathological process in various cardiovascular diseases [25]. In cardiac fibrosis, excessive collagen deposition and extracellular matrix accumulation result in myocardial hypertrophy, cardiac dysfunction, and arrhythmias [26–28]. The experimental results revealed that, in the TAC group, the gap between cells had widened and the residual number of myocardial cells decreased, showing overall fibrosis. Micrographs indicated that WXKL and KN93 improved the tissue morphology. The enhancement of collagen deposition plays an important role in adverse remodeling of the cardiac tissue [29]. Hence, stained sections were observed under a light microscope, and the results showed that KN93 and WXKL greatly decreased the degree of collagen deposition. The ultrastructure of cardiac myocytes showed that the myocardial cells in rats also showed slight recovery after KN93 and WXKL drug treatments. Thus, WXKL and KN93 improved cardiac fibrosis and decreased the degree of cardiac hypertrophy.

Cardiac fibrosis can not only cause cardiac hypertrophy but also affect cardiac function. It can also cause substitution of the myocardium with nonfunctional fibrotic tissue, thus leading to impaired ventricular systolic and diastolic functions, as well as atrial and ventricular arrhythmias and heart failure [30, 31]. Here, we evaluated cardiac function through a combination of echocardiography measurements that included the parameters LVPWS, FS%, and LVIDs. After treatment for 3 weeks, the LVPWS of the KN93 and WXKL groups were not any thinner than that in the TAC group. Interestingly, after treatment for 9 weeks, the LVPWS was significantly thicker in the KN93 and WXKL groups compared with the TAC group. These results showed that myocardial hypertrophy had formed after 3 weeks, and WXKL and KN93 had an effect of decreasing hypertrophy. However, the cardiac chambers were enlarged, and the myocardium became thinner in the TAC group after 9 weeks. After both 3 and 9 weeks of treatment, the ratios of HW/BW and PW/BW of the TAC group were higher than those of the Sham group, but the ratios of the KN93 and WXKL groups were less than that of the TAC group. The enlargement of the ventricle and increase in cardiac weight result in a decrease in cardiac function, thus resulting in cardiac decompensation [32]. After 3 weeks of treatment, the FS% decreased in the TAC group compared with the Sham group, but it increased in the WXKL group compared with the TAC group. After 9 weeks of treatment, the FS% of the KN93 and WXKL groups was significantly higher than that of the TAC group. However, the LVIDs of the TAC group were significantly different from those of the KN93 and WXKL groups after 3 weeks, but, after 9 weeks of treatment, the LVIDs decreased in the KN93 and WXKL groups compared with the TAC group. These results showed that WXKL and KN93 contributed to the improvement of cardiac function and inhibited the process of cardiac hypertrophy.

Proper functioning of the heart depends on normal action potential, which in turn depends on the normal functioning of ion channels. The abnormality that is most commonly found in animal models of cardiac hypertrophy is the prolongation of the APD [33–35]. It has been shown that CaMK II inhibition has little effect on APD in a transgenic mouse with increased endogenous CaMK II activity [36]. However, treatment with KN93 has been shown to contribute to the shortening of APD [37]. In our model, we found that the APD of the TAC group was significantly prolonged compared to that of the Sham group after 3 weeks, a result consistent with results from previous studies [38, 39]. After treatment with KN93 and WXKL, the APD_90_ changed slightly, but this change was not statistically significant. After 9 weeks of treatment, the results showed that WXKL and KN93 further shortened the APD_90_. Because some studies have reported the use of WXKL for the treatment of patients with arrhythmia, testing this parameter may be a reasonable and effective choice [39, 40]. Thus, this paper investigated the antiarrhythmic effects of WXKL and KN93 in the TAC model using an electrophysiological technology. Other studies have also demonstrated that increased CaMK II activity is linked not only to cardiomyopathy [41] but also to fatal arrhythmias, owing to the prolongation of APDs and development of premature depolarization.

CaMK II overexpression contributes to the development of heart failure [42], is associated with arrhythmias [43–46], and has detrimental consequences in irreversible ischemia and perfusion injury [47]. CaMK II is one of the most important regulators of intracellular Ca^2+^-cycling and acts through phosphorylation of various proteins such as RyR2 and PLB [48]. When CaMK II expression and activation are increased, RyR2 phosphorylation and the diastolic SR Ca^2+^ leakage are also increased [49]; this diastolic SR Ca^2+^ leak can initiate DADs in which the depolarizing current consists of an inward Na^+^/Ca^2+^ exchange [50]. However, dephosphorylated PLB inhibits SERCA2a activity, whereas the phosphorylation of PLB by cAMP-dependent protein kinase or CaMK II reverses SERCA2a inhibition, thus increasing Ca^2+^ uptake into the luminal SR [51]. In the present study, the protein expression of Thr-286 was significantly increased in the TAC group. WXKL and KN93 significantly decreased the level of Thr-286 in rats with TAC. WXKL and KN93 also increased the level of Thr-17 in rats with cardiac hypertrophy. The protein levels of RyR2 slightly recovered after WXKL and KN93 treatment, but there were no significant differences. Nevertheless, WXKL and KN93 also significantly increased the expression of Ser-2814. These possibly enhance the ability to modulate the CaMK II signal transduction pathway. This may be the most important mechanism by which WXKL and KN93 inhibit cardiac hypertrophy.

## 5. Conclusions

In summary, the present study evaluated the effects of WXKL and KN93 on the expression of CaMK II and on the electrophysiological parameters in TAC rats. The results demonstrated that the prolongation of APD_90_ resulting from cardiac hypertrophy was the electrophysiological mechanism causing cardiac arrhythmias, and KN93 and WXKL shortened the process and improved electrical remodeling. We also demonstrated that WXKL and KN93 significantly modulated the expression of CaMK II and improved the expression of related proteins in rats with arrhythmias. This may be the most important mechanism by which WXKL and KN93 inhibit cardiac hypertrophy. Although the precise mechanism is still not known, these findings may provide new clues for the improvement of clinical management by generating new targets for antiarrhythmic drugs from plants. Further in-depth research is required.
